# Prevalence of anxiety and depression symptoms and their associated factors in mild COPD patients from community settings, Shanghai, China: a cross-sectional study

**DOI:** 10.1186/s12888-018-1671-5

**Published:** 2018-04-04

**Authors:** Tian Xiao, Hua Qiu, Yue Chen, Xianfeng Zhou, Kang Wu, Xiaonan Ruan, Na Wang, Chaowei Fu

**Affiliations:** 1 Department of Epidemiology, School of Public Health; Key Laboratory of Public Health Safety; Pudong Institute of Prevention Medicine, Fudan University, Shanghai, 200032 China; 2 Department of Chronic Disease, Pudong New Area Center for Disease Control and Prevention, Shanghai, 200136 China; 3 School of Epidemiology and Public Health, Faculty of Medicine, University of Ottawa, Ottawa, ON K1G 5Z3 Canada

**Keywords:** Depression, Anxiety, Quality of life, COPD, Mild

## Abstract

**Background:**

Chronic obstructive pulmonary disease (COPD) is a serious disease frequently accompanied by anxiety and depression. Few studies have focused on anxiety and depression for mild COPD patients in China. This study aimed to assess the prevalence and associated factors for anxiety and depression among patients with mild COPD in urban communities.

**Methods:**

A cross-sectional survey of 275 mild COPD patients was conducted in 6 communities randomly sampled from Pudong New Area of Shanghai, China, in 2016. Data on socioeconomic factors and health conditions were acquired through a face-to-face interview as well as a physical examination. The Hospital Anxiety and Depression Scale (HAD) and EQ-5D visual analogue (EQ-5D_vas_) were applied to evaluate their mental health and quality of life, respectively. Logistic regression model was used to estimate adjusted odds ratios (aORs) and their 95% confidential intervals (CI) for risk factors associated with anxiety or depression.

**Results:**

Among 275 subjects, 8.1% had anxiety and 13.4% had depression. Logistic regression analysis indicated that female patients were more likely to suffer from anxiety than male patients (aOR = 6.41, 95% CI:1.73**-**23.80). Poor health status (EQ-5D_vas_ score < 70) was significantly associated with increased risks of anxiety (aOR = 5.99, 95% CI: 2.13-16.82) and depression (aOR = 2.67, 95% CI: 1.29-5.52).

**Conclusions:**

There were increased risks of anxiety and depression in mild COPD patients living in urban communities. Female sex and poor health status were significantly correlated to anxiety or depression. More interventions should be developed to reduce the risks of anxiety and depression at the early stage of COPD.

**Electronic supplementary material:**

The online version of this article (10.1186/s12888-018-1671-5) contains supplementary material, which is available to authorized users.

## Background

Chronic obstructive pulmonary disease (COPD) was the fifth leading cause of mortality globally in 2011, and was expected to be the third leading cause by 2030 [1]. In 2010, the total cost of COPD was 50 billion US dollars and the average cost per patient was over 4000 US dollars in the US, while they were respectively 36.9 billion and 2259 US dollars in China [2–6].

Patients of COPD are at a considerable risk of suffering from symptoms of depression and anxiety [7]. The prevalence of anxiety symptoms varied from 30 to 90%, and that of depressive symptoms ranged from 13 to 70% among COPD patients (mostly inpatients) [8–13]. Previous studies indicated that anxiety and depressive symptoms in COPD patients were correlated with various factors including age, gender, severity of COPD, general health status, smoking, physical endurance and social performance [14–18]. These studies were mainly conducted among inpatients who had severe COPD, and few of them focused on the prevalences of depression and anxiety and their influencing factors among mild COPD patients in community settings [8–19]. The aim of this study was to estimate the prevalences of anxiety and depression among mild COPD patients living in urban Chinese communities and their associated factors.

## Methods

### Study site and population

A cross-sectional study was carried out in Pudong New Area of Shanghai, China, from June to August in 2016. A register system including data of diagnosed COPD patients from all secondary and tertiary hospitals was created in 2014. Six out of 46 communities were selected randomly to recruit patient with mild COPD. At the enrollment, spirometry function test was implemented on each patient to verify their COPD severity. Inclusion criteria were as follows: 1) a person was previously diagnosed as mild COPD according to the Global Initiative for Chronic Obstructive Lung Disease (GOLD) (forced expiratory volume in one second of percent predicted (FEV1% predicted) ≥80%, the ratio of FEV1 to forced vital capacity (FVC) < 0.7) [20]; 2) a person provided informed consent and medical records were available; and 3) a person was a local resident aged 40 to 70 years and was capable of participating in the study by themselves. Patients with serious or unstable disease(s) (such as cardiovascular, neurological and musculoskeletal diseases) or inpatients were excluded. Patients with cognitive impairment and mobility limitation were excluded as well. Among 300 patients who participated in the study, 275 (91.7%) met all the criteria and were included in this analysis.

### Data collection and quality control

All information was collected by using a structured questionnaire and a physical examination. Demographic information included age, alcohol drinking history (Yes/No), smoking history (Yes/No), years of education (< 9 years/≥9 years), monthly household income (< 3000 RMB/≥3000 RMB), and regular exercise (Yes/No). Clinical information collected included disease duration, regular use of COPD medications in the past 12 months (Yes/No), exacerbation in the past 12 months (Yes/No) and comorbid conditions (hypertension, diabetes, kidney disease, stroke, cardiovascular diseases or others).

All field investigators received specific trainings in questionnaire interviewing, data recording and specific medical testing (scale assessment and spirometry function test) before the study was conducted. Questionnaire data were double-checked for its accuracy and completeness.

### Measurements of anxiety and depression symptoms as outcome variables

The Hospital Anxiety and Depression Score (HAD) is a concise and commonly used questionnaire to assess the existence of depression and anxiety symptoms for COPD patients [21–25] (Additional file 1). The HAD is a self-administered questionnaire with two subscales (HAD-A for anxiety symptom and HAD-D for depression symptom) and each subscale contains seven items. Each item score ranges from 0 to 3, and the total score for each subscale ranges from 0 to 21 (0 manifests rare symptom, and 3 indicates apparent symptom) [21, 22]. Studies showed that HADs with ≥8 points as cut-off points for anxiety and depression symptoms performed great sensitivity and specificity in Chinese patients [19, 26]. Participants with a HAD score greater than 8 points were more likely to suffer from anxiety or depression, and a higher score implies more advanced severity of clinically anxious or depressed symptom [15, 18, 21, 27].

In the current study, we defined a patient with “anxiety” or “depression” as having a HAD-A or HAD-D score of 8 points or higher.

### Spirometry function test, physical examination and health status assessment

Spirometry function test was performed at enrollment to acquire FEV1 and FVC values as well as FEV1% predicted to determine COPD severity or stage according to the guideline for COPD [20]. Each patient inhaled 20 ml salbutamol before pulmonary function testing was conducted and then complied with steps required for a spirometry function test. Final pulmonary function testing values were the average values of two satisfactory tests. Height and weight were measured to calculate body mass index (BMI, weight (kg)/height^2^ (m^2^)). In addition, health status was assessed with EQ-5D visual analogue (EQ-5D_vas_), which was considered to be a reliable and efficient tool for previous COPD studies [28–30]. Two previous studies used the visual analogue as a measure of quality of life in the association with depressive and anxious symptoms [31, 32]. For the EQ-5D_vas_ test, each patient drew a horizontal line on a scale to represent their health status, ranging from 0 (worst imaginable health status) at the bottom to 100 (best imaginable health status) at the top.

### Statistical analysis

Data analysis was performed by using SAS 9.2 for Windows (SAS Institute, Inc., Cary, NC). Pearson Chi square test or Fisher exact test was used for comparisons of categorical variables. Student t-test or Wilcoxon test was applied for two-group comparisons of continuous variables with or without a normal distribution. Logistic regression analysis was used to estimate crude odds ratios (cORs) and adjusted OR (aOR) as well as their 95% confidential intervals (CI) for risk factors associated with anxiety or depression. The *p* value of < 0.05 was considered statistically significant.

## Results

### Basic characteristics of subjects

Among 275 patients, 134 (48.7%) were male. The average age and BMI were 61.5 ± 6.0 years and 25.1 ± 4.0 (Kg/m^2^), respectively. Nearly 40% of participants had a history of smoking, with a higher proportion of ever smoking in male patients than in female patients. Women had a longer average duration of COPD (13.2 years) and were more likely to have a comorbid condition (59.6%), compared to men. There were no significant differences in EQ-5D_vas_ scores, regular use of COPD medications, as well as exacerbation in the past 12 months between male and female patients (Table 1).

**Table 1:** Basic information among male and female patients with mild COPD

Characteristics	Total	Male	Female	*P* value
Number [*n*(%)]	275	134(48.7)	141(51.3)	.
Age, years [mean(SD)]	61.5(6.0)	62.1(5.4)	60.9(6.5)	0.113
BMI (kg/m2) [mean(SD)]	25.1(4.0)	24.7(3.9)	25.5(4.2)	0.074
Ever alcohol drinking [*n*(%)]	54(19.6)	49(36.6)	5(3.5)	**< 0.0001**
Ever smoking [*n*(%)]	115(41.8)	113(84.3)	2(1.4)	**< 0.0001**
Regular exercise [*n*(%)]	98(35.6)	48(35.8)	50(35.5)	0.95
Years of education [*n*(%)]				
< 9	229(83.3)	109(81.3)	120(85.1)	0.403
≥ 9	46(16.7)	25(18.7)	21(14.9)	
Monthly household income per capita/Yuan [*n*(%)]				
< 3000/per month	141(51.3)	57(42.5)	84(59.6)	**0.005**
≥ 3000/per month	134(48.7)	77(57.5)	57(40.4)	
Duration of COPD, year [mean(SD)]	11.3(14.1)	9.4(13.9)	13.2(14.1)	**0.004**
EQ-5Dvas score [mean(IQR)]	70.6(14.3)	70.1(14.6)	71.0(14.1)	0.322
Regular COPD medication use, in recent 12 months [*n*(%)]	97(35.3)	48(35.8)	49(34.8)	0.853
Exacerbation, in recent 12 months [*n*(%)]	77(28.0)	35(26.1)	42(29.8)	0.498
Comorbidity [*n*(%)]	145(52.7)	61(45.5)	84(59.6)	**0.020**

*Abbreviations: BMI* body mass index

For comparison, χ^2^ test used for binary variables, and Student’s t test or Wilcoxon non-parametric test employed for continuous variables; the bold *P* values indicated the statistical significance

### Prevalences of anxiety and depression

**Fig. 1:**
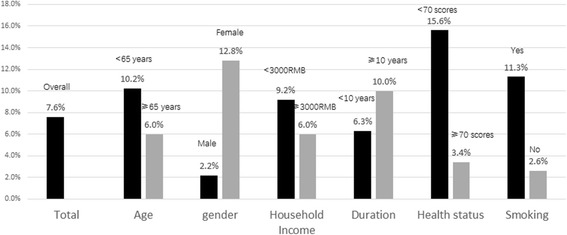
Prevalence of anxiety symptoms among patients of mild COPD

**Fig. 2:**
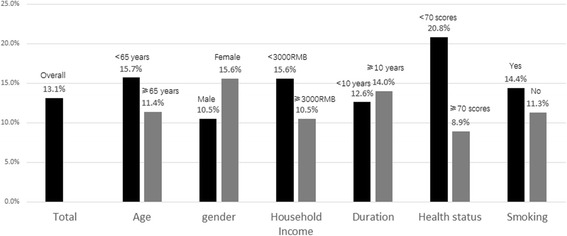
Prevalence of depression symptoms among patients of mild COPD

The overall prevalences of anxiety and depression were 7.6 and 13.1%, respectively. Figure 1 shows the prevalences according to sex, smoking history and health status. An increased risk of anxiety was observed in female patients (12.8%, *P* < 0.001), smokers (11.3%, *P* = 0.008) and those with poor health status (EQ-5D_vas_ score < 70) (15.6%, *P* = 0.0003). Patients with poor health status (EQ-5D_vas_ score < 70) (20.8%) were more likely to suffer from depression than those with better health status (8.9%) (*P* = 0.005) (Fig. 2).

### Factors associated with anxiety and depression

After adjustment for covariates, female patients were more likely to suffer from anxiety (aOR = 6.41, 95% CI: 1.73-23.80), and patients with poor health status (EQ-5D_vas_ score < 70) had a higher risk of anxiety (aOR = 5.99, 95% CI: 2.13-16.82) (Table 2). Similarly, poor health status was significantly associated with an increased risk of depression (aOR = 2.67, 95% CI: 1.29-5.52) after important covariates were taken into account (Table 3).

**Table 2:** Possible factors associated with anxiety symptoms among mild COPD patients

Characteristics	cOR(95%CI)^a^	aOR(95%CI)^b^
Gender		
Male	1.00	1.00
Female	**6.39(1.84~ 22.23)**	**6.41(1.73~ 23.79)**
Age, years		
< 65	1.00	1.00
≥ 65	0.56(0.23~ 1.37)	0.58(0.21~ 1.55)
Household income per capita/Yuan		
< 3000/per month	1.00	1.00
≥ 3000/per month	0.86(0.63~ 1.16)	1.04(0.74~ 1.46)
Duration, years		
< 10	1.00	1.00
≥ 10	1.66(0.68~ 4.05)	1.21(0.45~ 3.19)
EQ-5Dvas score		
≥ 70	1.00	1.00
< 70	**5.34(1.20~ 14.27)**	**5.99(2.13~ 16.82)**
Comorbidities		
No	1.00	1.00
Yes	0.98(0.40~ 2.40)	0.90(0.33~ 2.43)

*Abbreviations: cOR* crude odds ratio, *aOR* adjusted odds ratio

^a^ Data represents univariate association as determined by logistic regression model

^b^ Variable included in the multivariate logistic regression model: gender, age, monthly household per capita, disease duration, EQ-5Dvas score and comorbidity; the bold OR (95% CI) indicated the statistical relationship

**Table 3:** Possible factors associated with depression symptoms among COPD patients

Characteristics	cOR(95%CI)^a^	aOR(95%CI)^b^
Gender		
Male	1.00	1.00
Female	1.58(0.77~ 3.24)	1.48(0.69~ 3.19)
Age, years		
< 65	1.00	1.00
≥ 65	0.69(0.34~ 1.39)	0.68(0.32~ 1.41)
Household income per capita/Yuan		
< 3000/per month	1.00	1.00
≥ 3000/per month	0.86(0.68~ 1.09)	0.91(0.71~ 1.17)
Duration, year		
< 10	1.00	1.00
≥ 10	1.13(0.55~ 2.33)	0.95(0.44~ 2.02)
EQ-5Dvas score		
≥ 70	1.00	1.00
< 70	**2.68(1.32~ 5.46)**	**2.67(1.29~ 5.52)**
Comorbidities		
No	1.00	1.00
Yes	0.88(0.44~ 1.78)	0.89(0.43~ 1.86)

*Abbreviations: cOR* crude odds ratio, *aOR* adjusted odds ratio

^a^ Data represents univariate association as determined by logistic regression model

^b^ Variable included in the multivariate logistic regression model: gender, age, monthly household per capita, disease duration, EQ-5Dvas score and comorbidity; the bold OR (95% CI) indicated the statistical relationship

## Discussion

In this cross-sectional study, the prevalence was 7.6% for anxiety and 13.1% for depression (13.1%) among mild COPD patients living in urban community. The prevalences for COPD patients were higher as compared with those for general populations [19, 33–35]. Among the general adult population in China, the prevalences of anxiety and depression were 5.3 and 7.2%, respectively, when assessed by the HADs scale, and were 5.6 and 6.1% when assessed by the Diagnostic and Statistical Manual of Mental Disorders fourth edition (DSM-IV) scale [19, 29]. Another Chinese cohort study of 0.5 million adults found that the prevalence of depressive symptoms was as low as 2.4%, which was similar to the finding from a meta-analysis of 21 studies covering 11 provinces [30, 33]. The prevalence of anxiety varied from 10 to 100%, while that of depression varied from 7 to 79% depending on the stage of COPD [34]. Most previous studies, which recruited COPD patients with moderate to extremely severe stages from hospitals, found inconsistent relationships between severity of COPD and mental health [19, 35–38]. Different instruments measuring anxiety and depression symptoms were used in different studies, which is one reason for study discrepancies [39, 40]. In this study, only mild COPD patients from community settings were included. Our prevalence estimates were lower than those using the same scale of HAD (9.6 to 49% for anxiety and 22.8 to 52% for depression) [11, 38] except for one that included patients with mild COPD only [19].

For COPD patients, women were more likely to suffer from anxiety, and poor health status assessed by using EQ-5D was associated with increased risks of both anxiety and depression. A systematic review of 10 studies showed that women were significantly more likely to suffer from anxiety than men (56% vs 35%, *P* = 0.04), and similar sex disparity also existed in other studies [36, 40, 41]. One possibility would be that female patients with COPD had a lower income level, a longer duration and more comorbid conditions, compared to male patients. However, sex difference remained significant after taking these factors into consideration. Similar finding for the relationships between health status and anxiety/depression were observed in other studies [14, 42, 43]. In one hand, those who have positive symptoms of anxiety or depression in general tend to report worse health status. In another hand, those with worse health status worry more about their conditions, which could worsen their anxiety or depression symptoms. A cross-sectional study provided no information on the direction of the associations. Therefore, cohort study design is required to further explore the nature of these associations.

There are strengths and limitations in this study. This study was conducted in urban communities and all invited subjects participated in the study. All subjects included in the study were confirmed to be at the mild stage of COPD based on a free post-bronchodilator pulmonary function test. However, a cross-sectional study design provided no evidence for causal associations between risk factors and presence of psychological symptoms. Since severe COPD cases were not included in this study and we could not assess the overall occurrences of anxiety and depression for COPD. In addition, the exclusion of any inpatients might result in an underestimation of the prevalences of anxiety and depression in mild COPD patients.

## Conclusions

In conclusion, anxiety and depression were common in patients with mild COPD living in urban communities and mental health care services should be provided to these patients. Female sex and poor health status were significantly correlated to anxiety and depression symptoms. More interventions should be developed to reduce the risks of anxiety and/or depression at the early stage of COPD in community settings.

## Additional file

Additional file 1: The questionnaire for COPD patients in communities of Pudong District, Shanghai. The questionnaire covered on data of geographic, socioeconomic, lifestyle and occupational exposure factors, and COPD and comorbidities. (PDF 288 kb)

## Abbreviations

CI: Confidence interval
DSM-IV: Diagnostic and statistical manual fourth edition
HAD: The Hospital Anxiety and Depression Score
IQR: Interquartile range
OR: Odds ratio
SD: Standard deviation
