# Supplementary material for: Prevalence of anxiety and depression symptoms and their associated factors in mild COPD patients from community settings, Shanghai, China: a cross-sectional study
Source: BMC Psychiatry. 2018 Apr 4;18:89. doi: 10.1186/s12888-018-1671-5 (PMC5883260; doi:10.1186/s12888-018-1671-5)
Supplement: Supplementary file 1 — The questionnaire for COPD patients in communities of Pudong District, Shanghai. The questionnaire covered on data of geographic, socioeconomic, lifestyle and occupational exposure factors, and COPD and comorbidities. (PDF 288 kb) [file 12888_2018_1671_MOESM1_ESM.pdf]

The questionnaire for COPD patients in communities of Pudong District, Shanghai

Investigation Date：_____year__month__day

Beginning time：____hour____minute

Name of investigator：_________

Note：Please choose the relevant item with “√”, or fill in the blank.

Geographic information

1. Name：_________

2. Sex：①Male ②Female

3. Birthdate：______year___month___day

4. Nationality：______

5. Tel：_________________

6. Identification number：_________________

7. Home Address：____

8. Status of marriage：①Married ②Unmarried ③Divorced ④Bereavement ⑤Separated ⑥Other

9. Education years：①0-6 ②7-9 ③10-12 ④13-15 ⑤16 or more

10. Occupation：①Workman ②Farmer ③Army man ④Officer ⑤Professional ⑥Staff ⑦Retired ⑧Unemployed ⑨Other________

11. Household income of per capita monthly (RMB)：

①<1000 ②1000~ ③3000~ ④5000~ ⑤>=8000 ⑥Unknown

12. Medical Insurance (For multiple choices)：

①None ②Basic medical insurance for urban staffs ③Basic medical insurance for urban residents ④Comprehensive insurance for immigrate employees ⑤New rural cooperative medical insurance ⑥Commercial medical insurance ⑦Other_______

Disease-related information

13. Date of COPD diagnosis：______ year ___month

14. Comorbidities (For multiple choices)：

①Other pulmonary diseases：□Pneumonia □Chronic bronchitis

□Emphysema □Asthma □Pulmonary cancer □Other______

②Hypertension ③Diabetes ④Kidney disease ⑤Heart disease ⑥ Other______

⑦None ⑧Unknown

15. COPD family history in 1-degree relatives (For multiple choices)：

①None ②Father ③Mother ④Brother (Yes:___/No:___) ⑤Sister (Yes: /No:___) ⑥Son (Yes:___/No:___) ⑦Daughter (Yes:___/No:___) ⑧ Unknown

16.Do you take drugs for COPD regularly in recent one year?

①Yes ②No, occasionally ③Never (Go to question 18, please)

17.Which kinds of the followings are taken by you? (For multiple choices)

①Inhaled β 2 receptor agonist ②Oral β 2 receptor agonist ③M choline receptor antagonist (inhalant) ④Corticosteroids ⑤Expectorants ⑥ Phosphodiesterase inhibitor ⑦Other___

18.Numbers of COPD exacerbation in recent one year：_______

19. Did you see doctor for COPD exacerbation in recent one year?

①Yes ②No

20. Complications (For multiple choices)：

① Spontaneous pneumothorax ② Pulmonary arterial hypertension ③ Chronic pulmonary heart disease ④ Venous thromboembolism ⑤ Respiratory insufficiency or failure ⑥Other________ ⑦None

21. Do you know about your lung function?

①Yes ②No

22. How often do you test your lung function?

① twice or more every year ② once every year ③ once every 2-years ④ once every 3-years ⑤ once over 4 or more years ⑥ None

23. Why not to have a lung function test? (For multiple choices)

① Unknown ② No suggestion from Doctor ③ No devices for that ④ Not necessary ⑤ Other___

Lifestyle and occupation exposures

24. Do you smoke now？

①No

②Yes, years of smoking:____，average number of smoking daily:_____， age of beginning smoking:____

③Smoking cessation, years of smoking cessation:___， years of smoking before cessation:___，average number of smoking daily before cessation _____，age of stopping smoking: _____

(Smoking refers continuous smoking at least one cigarette daily for more than

six months；smoking cessation refers to continuous smoking at least one cigarette daily for more than six months in the past，but now has completely stopped smoking)

24. How often have you been exposed to second-hand smoke？

①Never ②Occasionally ③Frequently, exposure years:___, average exposure hours daily: _____

(Second-hand smoking defined as that non-smokers inhale cigarette smoke from smokers lasting for 15 minutes or longer.)

25.Do you drink any alcohol?

①Never

②Beer, years of drinking:____, average times of drinking weekly:___, average volume of drinking: _____

③Yellow wine, years of drinking:____， average times of drinking weekly:___, average volume of drinking: _____

④Liquor, years of drinking:____， average times of drinking weekly:___, average volume of drinking: _____

⑤Wine, years of drinking:____, average times of drinking weekly:___, average volume of drinking: _____

⑥Others______, years of drinking:____， average times of drinking weekly:___， average volume of drinking: _____

⑦Cessation, years of drinking:____， average times of drinking weekly:___, average volume of drinking: _____

26.Have you being working in certain condition with dust，asbestos，pernicious smoke or harmful gas for one year at least？ ①Yes ②No

27. Which heating equipment do you have at home？

①Air conditioner ②Heating underground ③Never ④Other________

28. Do you use fresh air ventilation system usually?

①Yes, from___Year___Month ②No

29.Do you use kitchen ventilator usually? ①Yes ②No

30. How often do you have physical exercises for half hour or longer?

① No ② <once weekly ③ once or twice weekly ④ 3 times or more weekly

31. Which kind of the followings do you have mostly?

①Walking ②Jogging ③Dancing ④Wushu ⑤Swimming

⑥Play football or basketball or other ballgames ⑦ Other_____

Thank you for your cooperation！

Terminal time： ____hour____minute

Investigator signature： _______________
